# Jin-Tian-Ge ameliorates ovariectomy-induced bone loss in rats and modulates osteoblastogenesis and osteoclastogenesis in vitro

**DOI:** 10.1186/s13020-022-00627-2

**Published:** 2022-10-05

**Authors:** Yi Shen, Na Wang, Qi Zhang, Yuling Liu, Qudi Wu, Yuqiong He, Yang Wang, Xiaoyan Wang, Qiming Zhao, Quanlong Zhang, Luping Qin, Qiaoyan Zhang

**Affiliations:** 1 School of Pharmaceutical Sciences, Zhejiang Chinese Medical University, Binwen Road 584, 310053 Hangzhou, People’s Republic of China; 2 Ginwa Enterprise (Group) INC, Xi’an, 710069 China; 3 Institute of Chinese Materia Madica, Shanghai University of Traditional Chinese Medicine, Shanghai, 201203 China; 4 Zhejiang Traditional Chinese Medicine & Health Industry Group CO., LTD, Hangzhou, 310016 China

**Keywords:** Jin-Tian-Ge, Osteoporosis, Osteoblast, Osteoclast, BMP and Wnt/β-catenin pathway, NF-κB pathway

## Abstract

**Background:**

Tiger bone, which had been one of the most famous traditional Chinese medicine for 2000 years, was originate from the skeleton of *Panthera tigris* L., and had the actions of anti-inflammatory, analgesic, immune-regulatory and promoting healing of bone fracture, and was used for the treatment of osteoporosis and rheumatoid arthritis. Jin-Tian-Ge (JTG), the artificial tiger bone powder, were prepared from skeletons of several farmed animals to substitute the natural tiger bone, and has been used for the treatment of osteoporosis in clinical practice. However, the characteristic and mechanism of action of JTG for the therapy of osteoporosis need to be further evidenced by using modern pharmacological methods. The aim of this work is to investigate the bone-protective effects of JTG, and explore the possible underlying mechanism.

**Methods:**

Ovariectomy (OVX) rats were orally administrated JTG or estradiol valerate (EV) for 12 weeks. We investigated the pharmacodynamic effects of JTG on anti-bone loss in OVX rats, and also investigated the role of JTG in promoting osteogenesis and inhibiting osteoclast differentiation.

**Results:**

JTG increased the bone mineral density (BMD), improved the bone microarchitecture and biomechanical properties in ovariectomized rast, whereas reversed the bone high turnover in OVX rats as evidenced by serum biochemical markers in OVX rats. JTG increased osteogenic differentiation of BMSCs in vitro, and up-regulated the expression of the key proteins of BMP and Wnt/β-catenin pathways. JTG also inhibited the osteoclastogenesis of BMM as evidenced by the alteration of the TRAP activity, F-actin construction and the expression of nuclear factor of activated T-cells cytoplasmic 1 (NFATc1), c-Fos, Cathepsin K (Ctsk) and matrix metallopeptidase 9 (MMP9) of OCs induced with RANKL and LPS, reduced the expression and phosphorylation of NF-κB in OCs.

**Conclusions:**

JTG prevented bone loss in OVX rats and increased osteogenic differentiation of BMSCs through regulation of the BMP and Wnt/β-catenin pathway, inhibited osteoclastogenesis by suppressing the NF-κB pathway, suggesting that JTG had the potentials for prevention and treatment of osteoporosis by modulating formation and differentiation of osteoblast and osteoclast.

**Supplementary Information:**

The online version contains supplementary material available at 10.1186/s13020-022-00627-2.

## Background

Osteoporosis, which is a common and frequently occurring diseases for the aged people, are characterized by decreased bone mass, deterioration of bone micro-architecture, and fragility to bone fracture [1–3]. This disease has attracted more attention of governments and social, because of its increased incidence rate, mortality and disability rate, and the high treatment cost and lowered quality of life [4]. Tiger bone, which was originated from the skeleton of *Panthera tigris* L., is one of the most famous traditional Chinese medicine, and traditionally described as having the efficacy of strengthening kidney and benefiting essence, strengthening bones and tendons, and has been used for the treatment of osteoarthritis, rheumatoid arthritis and osteoporosis [5–8]. The chemical analysis and pharmacological investigation demonstrated that tiger bone is abundant in minerals (calcium, phosphorus, magnesium, iron and zinc, etc.), collagen and amino acid, and has been showed to possess the effects of anti-inflammatory, analgesic, immune-regulatory and promoting healing of bone fracture [6, 7, 9]. However, according to Convention on International Trade in Endangered Species (CITES), *Panthera tigris* L., as endangered animal, should be protected, and hence the tiger bone has also been banned to use for medicinal purpose in China since 1993 [10]. In order to meet the medical demand, Jin-Tian-Ge (JTG), the artificial tiger bone powder, has been developed to substitute the natural tiger bone. JTG, which were prepared from skeletons of several farmed animals, such as goat, deer, etc., has similar chemical composition and pharmacological actions with natural tiger bone [6]. The pharmacological investigation revealed that JTG possess anti-inflammatory, analgesic, and anti-osteoporotic activities [11–13]. JTG Capsule can improve the bone density of lumbar spine and femoral neck in patients with primary osteoporosis [12], also can increase the levels of blood calcium, bone density, bone strength and toughness, and reduce bone resorption in retinoic acid-induced osteoporotic rats [14]. Furthermore, JTG capsule increase the activities of alkaline phosphatase (ALP) and osteocalcin (OCN) of osteoblast, decrease the production of pyridinoline (PYD) in osteoclast [15]. JTG has been approved by the China Food and Drug Administration (CFDA; China, Z20030080) for the treatment of osteoporosis and osteoarthritis [12, 13, 16]. The clinical trials demonstrated that JTG alone, or combined with other anti-osteoporotic medications showed significant potentials in improving BMD, relieving pain, and reducing adverse events of other chemical drugs. Recently, a multicenter, double blind, placebo-controlled, and dose–effect clinical trial further confirmed the safety and efficacy of JTG in the treatment of osteoporosis [11]. However, research on the characteristic and underlying mechanism of JTG for the therapy of osteoporosis was lacking.

The primary osteoporosis is often caused by either postmenopausal estrogen loss or age-related oxidative stress. Estrogen exerts protective effects on skeleton in both rodents and humans. A decrease in the estrogen level associated with menopause leads to a decrease in bone mineral density (BMD) and deterioration of bone microarchitecture [17]. The ovariectomized rats are often used to simulate osteoporosis related to estrogen deficiency. Ovariectomy decreases the levels of estrogen, and causes bone loss, leading to degradation of bone matrix and an increased levels of DPD and CTX-I in urine and serum of rats. This rapid bone loss is featured with an increase in bone turnover with an imbalance between bone resorption and bone formation [18]. Hence, the serum levels of ALP, OCN and PINP exhibiting activities of bone formation and serum levels of TRAP, RANKL and OPG indicating activities of bone resorption were used to evaluate the status of bone metabolism in OVX rats, and are often used to assess the protective effects of anti-osteoporotic medications on bone loss [19].

Bone homeostasis is maintained through a balance between osteoblastic bone formation and osteoclastic bone resorption, and excessive osteoclastic activity and inadequate osteoblastic activity may lead to bone loss, and then the incidence of osteoporosis [2, 20]. Osteoblast are differentiated from bone marrow mesenchymal stem cell (BMSC) under the control of BMP and Wnt/β-catenin pathway, while osteoclast are derived from bone marrow macrophages (BMMs) under the regulation of transcription factor c-fos, NFATC1 and NF-ƙB pathway [21–23]. Therefore, the medical therapy of osteoporosis includes inhibition for osteoclast driven bone resorption, and increase for osteoblast driven bone formation and mineralization. To verify the efficacy and explore the underlying mechanism of JTG in prevention and treatment of osteoporosis, the present study was designed to evaluate the antiosteoporotic effects of JTG in ovariectomized rats, and investigate its regulatory effects on differentiation of osteoblast derived from BMSCs and osteoclast from BMMs, with the hope to provide further evidence for its clinical application.

## Materials and methods

### Reagents and animals

Jin-Tian-Ge (JTG) were prepared from skeletons of several farmed animals, including *Cervus elaphus* Linnaeus, *Cervus nippon* Temminck, *Capra hircus* Linnaeus, and *Sus scrofa domesticus* Linnaeus by Ginwa Enterprise Group INC, Xi’an, China, and their ingredients includes 18% calcium, 8% phosphorus, peptides and proteins.

The regents used in this study were estradiol valeratse (E_2_V, Bayer Health Care Co., Ltd., Guangzhou, China); penicillin (Shandong Lukang Pharmaceutical Co., Ltd., Shandong, China); the bio-markers for bone matebolism including commercially enzyme linked immunosorbent assay (ELISA) kit of deoxypyridinoline cosslinks (DPD), procollagen I N-Terminal propeptide (PINP), osteoprotegerin (OPG), osteocalcin (OCN), C-Telopeptide of type I collagen (CTX-I), tartrate resistant acid phosphatase (TRAP), receptor activator of nuclear factor kappa B ligand (RANKL), and biochemical kit of calcium (Ca) and alkaline phosphatase (ALP) (Nanjing Jiancheng Bioengineering Institute, Nanjing, China); All other reagents were of analytical-grade purity, and purchased from Sinopharm Chemical Reagent Co., Ltd.

Fetal bovine serum (FBS), α-Modified minimal essential medium (α-MEM), phosphate buffered saline (PBS) and penicillin/streptomycin were obtained from Gibco company (USA). Ascorbic acid, β-glycerophosphate, dexamethasone, Acid Phosphatase Kit, 6-diamidino-2-phenylindole (DAPI) were purchased from Sigma-Aldrich (St. Louis, MO, USA). Receptor activator of nuclear factor κB ligand (RANKL) and macrophage colony-stimulating factor (M-CSF) were purchased from Peprotech (EC, USA). Antibodies against BMP2 and CtsK were purchased from Abcam (Cambridge, MA, USA). Antibodies against Wnt3a, IkBα, c-Fos, MMP9 and TRAF6 were purchased from Boster Biological Technology (Wuhan, China). Antibodies against Smad4, Smad1/5/9, Runx2, LRP5, β-catenin, P-β-catenin, glycogen synthase kinase-3 β (GSK-3β) and p-GSK-3β, p65, p-p65, NFATc1 and GAPDH were obtained from Cell Signaling Technology (Beverly, MA, USA). The BCA Protein assay kits was from Biyotime (Shanghai, China).

Sixty female Wistar rats ages 10 weeks were purchased from Sippur Will Kay Company and housed at the Experimental Animal Center of Zhejiang Chinese Medicine University (Hangzhou, China, Certificate No. SYXK 2018-0012). The rats were acclimatized on a 12 h light–dark cycle under a temperature of (24 ± 0.5) ℃ and humidity of (47.5 ± 2.5)%. All animals were handled according to National Institute of Health (NIH) guidelines on the ethical use of animals, and received humane care.

### Determination of amino acid contents in JTG

The samples of JTG were treated according to hydrolysis method of proteins provided in protocols of Hitachi L-8900 Amino Acid Analyzer. The amino acid in JTG were determined by using Hitachi L-8900 Amino Acid Analyzer (Tokyo, Japan). The chromatographic column is 4.6 mm I.D. × 60 mm L packed with Hitachi custom ion exchange resin (Partical size: 3 µm). The detective wavelength were 570 nm and 440 nm. The elutes and gradient elution procedures were in accordance with the protocols of this instrument.

### Animal experimental protocol

#### Grouping and drug administration of rats

Sixty rats were equally randomized into six groups, with ten rats in each group. The rats in Sham group were subjected to cut off some fat tissue close to the ovaries, and the other fifty rats were removed bilateral ovaries as previously described (OVX), and randomly divided into five groups, including OVX model group, positive control group and low, middle and high dose of JTG treatment groups [19]. One week after ovariectomy, the rats in Sham and OVX group were orally administered with distilled water containing 0.5% CMC-Na (1 mL/100 g body weight), the OVX rats in positive control group were orally administered with E_2_V (0.2 mg/kg body weight, once every day), the OVX rats in JTG treatment groups were orally administered with different doses of JTG (180, 360 and 720 mg/kg body weight, once every day), respectively. The rats were treated orally with vehicle, E_2_V and JTG for 12 weeks. The body weight of rats was weighed every week to adjust the dosage of E_2_V and JTG. At the end of the experiment, all rats were sacrificed by cervical dislocation after serum samples collection. The uterus was immediately removed and weighed. This experiment was approved by the Bioethic Committee of the Zhejiang University of Traditional Chinese Medicine (Approval NO. IACUC-20190311-09), and the procedures of the experiment were strictly according to generally accepted international rules and regulations.

### Determination of bio-marker levels in serum and urine of rats

Rat blood was collected and allowed to curdle 2 h at room temperature. Serum was collected and frozen at – 80 °C for biochemical markers assay. The levels of bio-markers related with bone metabolism including OCN, OPG, PINP, CTX-I, RANKL, TRACP and ALP were blindly measured with ELISA and biochemical kits following the standard kit procedure.

Rats in each group were placed in metabolic cages before sacrifice, and fasted and free drink water, and the urine were collected for 12 h. Urine was frozen at – 80 °C for biochemical markers assay. The biomarker levels of bone metabolism including DPD and Ca were measured with ELISA and biochemical kits according to the kit protocols.

### Micro-CT analysis

The right femur of rats was removed, and then fixed in 4% paraformaldehyde for more than 24 h. The BMD and parameters of bone histomorphometry at distal femoral were blindly analyzed with micro-CT (V1.5.22, Skyscan1172, Belgium). The analysis conditions were as follows: the scanner resolution was 9 μm, with source current of 112 μA, and source voltage of 80 kV. Rotation step was 0.6 degree, exposure time 370 ms, and the frame averaging was 2. Image resolution ratio was 17.81 µm. Three-dimensional reconstruction was conducted with the NRecon analysis workstation. One millimeter below the center of the epiphyseal line was selected as the region of interest (ROI) for the analysis. The parameters of bone histomorphometry were obtained, including bone mineral density (BMD), bone surface to bone volume (BS/BV), relative bone volume (BV/TV), trabecular separation (Tb.Sp), trabecular thickness (Tb.Th), and trabecular number (Tb.N).

### Three point bending test

The left femur of rats was removed and preserved in – 20 °C. The femurs were thawed to room temperature, and to determine the length, width in long axis and width in short axis using Vernier Caliper. The three point bending test were performed by using Universal Material Testing Machine (Instron 5569). The analysis conditions were as follows: the crosshead speed was 1 mm/min, with span length of 15 mm, and the size of load cell of 1000 N. The sample size was measured by UTM according to the description in literature [24]. The parameters of bone biomechanics including bone material toughness, bending stiffness, maximum load, elastic modulus, maximum strain and maximum stress were obtained by calculating as follows.

Stress (σ, N/mm^2^) is typically defined as force per unit area, and strain (ε, mm/mm) is typically defined as percentage change in length, or relative deformation [25]. The load can be converted to stress and deformation through engineering formulae, the relationship between stress and strain in bone follows a curve called the stress–strain (σ-ε) curve. The slope of the stress–strain curve within the elastic region is called the elastic modulus (E) [26]. Elastic modulus (E), which is the amount of energy per unit volume necessary to cause damages as well as permanent compositional and structural changes of bone, is calculated from the ratio of stress/strain (σ/ε) [27]. Stress, strain, and elastic modulus can be calculated from the force (F) and displacement (d) of the loaders. For three point loading, the equations are

1 $$\sigma = \frac{{{\text{FLc}}}}{{4{\text{I}}}} , \, \varepsilon = \frac{{12{\text{cd}}}}{{{\text{L}}^{2} }},\,\,{\text{and}}\,{\text{E}} = \frac{\sigma }{\varepsilon } = \frac{{\text{F}}}{{\text{d}}}\frac{{{\text{L}}^{{3}} }}{{48{\text{I}}}},$$

where c is the distance of force point to the center of mass; L is the length between the two fulcrum; I is cross-sectional moment of inertia around the axis of bending, can be estimated for the cross-section of a long bone by using formulae for a perfectly elliptical cross-section [24, 26, 28, 29]. Maximum load, which is the force that cause a specimen broken at maximum height of the curve, were directly measured from the load–displacement diagram [30]. For bending tests, the intrinsic stiffness of the bone is equal to the elastic modulus (E); and the extrinsic stiffness or bending stiffness is equal to E * I. Furthermore, the bone material toughness, also called as energy absorption, can be derived from the area underneath the stress–strain curve [25, 29]. Maximum stress (σ, N/mm2) and maximum strain (ε, mm/mm) is force per unit area and percentage change in length, respectively, when the bone is broken [25].

### Analysis of protein expression in bone tissue of rats

The left femur in hind limbs of rats were collected, and the muscle and excess tissues were removed, the bones marrow were flushed with PBS solution using a syringe. The bone of rats was ground in liquid nitrogen. The proteins of bone tissue were extracted using 200 μL lysate containing phosphatase inhibitors, protease inhibitors, PMSF and IP lysis buffer (v/v 20: 20: 10: 950) in a 1.5 mL eppendorf tube on the ice for 20 min. The extracted mixture was centrifuged (12,000 rpm) at 4 °C for 20 min, and the supernatant containing proteins of bone tissue were transferred into a 1.5 mL Eppendorf tube. The proteins were quantified and denatured for Western-blot analysis.

### Osteoblast experimental protocol

#### BMSC culture and identification

BMSCs were isolated from the femur of the 3-week-old Sprague Dawley rat, and cultured in a dish containing α-MEM supplemented with 10% FBS, 100 U/mL penicillin, 100 mg/mL streptomycinn and 1% l-Glutaminea in humidified atmosphere of 5% CO_2_ for 10 days. The medium was changed every 3 days. The subculture cells (passage 2–4) were used for subsequent experiments. BMSCs were identified in terms of the morphological character, properties of differentiation into osteoblasts and adipocytes, and expression of CD29 and CD45. This experiment was approved by the Bioethic Committee of Zhejiang University of Traditional Chinese Medicine.

#### Cell proliferation assay

BMSCs were seeded in a 96-well culture plate at a density of 5 × 10^4^ cells per well and cultured in α-MEM medium with 10% FBS, treated with different concentrations of JTG for 48 h. Cell proliferation was measured by CCK-8 assay. 10 µL CCK-8 solution was added to each well and then incubated for appropriate time. The absorbance was determined at 450 nm with a microplate reader.

#### ALP staining and activity determination

BMSCs were seeded in a 48-well culture plate at a density of 2.5 × 10^5^ cells per well and cultured in α-MEM medium with 10% FBS, and treated with different concentrations of JTG for 7 days to perform ALP staining and determination of ALP activity. The cells were stained with BCIP/NBT assay kit at 37 °C for 30 min. ALP activity was measured according to the manufacturer’s instructions of the assay kit. Briefly, the cells were washed twice with PBS, and then lysed on ice for 30 min. The cell lysate was collected by centrifuge, and mixed with detection buffer and color substrate, and then incubated for appropriate time. The absorbance was determined at 405 nm with a microplate reader. The alkaline phosphatase activity was calculated according to the definition of enzyme activity provided in the instructions of the assay kit.

#### Alizarin red staining for analysis of bone mineralized nodule formation

BMSCs were seeded in a 48-well culture plate at a density of 2.5 × 10^5^ cells per well and cultured in osteogenic medium (50 µg/mL ascorbic acid, 10 mM sodium β-glycerophosphate and 10^–8^ M dexamethasone) and treated with different concentrations of JTG for 21 days. Then, cells were fixed with 4% paraformaldehyde and stained with alizarin red (pH 8.3) at 37 °C for 30 min. Bone nodules were observed under a microscope. To quantify cell mineralization, bone nodules were dissolved with 10% (V/W) cetylpyridinium phosphate chloride, and the absorbance was measured at 570 nm with a microplate reader.

#### The analysis of expression of protein involved into osteoblast differentiation and activities

BMSCs were seeded in a 6-well culture plate at a density of 1 × 10^6^ cells per well and cultured in α-MEM medium with 10% FBS, and then treated with different concentrations of JTG for 7 days. Subsequently, cells were lysed and the protein content was determined using a BCA protein kit. The western blot analysis was used to determine the expression of proteins.

### Osteoclasts experimental protocol

#### Osteoclasts culture

C57BL/6 mice aged 6 weeks were used to isolate bone marrow macrophages (BMMs) from the femur. The extracted bone marrow cells were cultured in α-MEM containing with 10% FBS, 100 U/mL penicillin, 100 mg/mL streptomycin and 5 ng/mL M-CSF in a humidified atmosphere of 5% CO_2_ for 24 h. Non-adherent cells, which were considered as BMMs were collected and cultured in α-MEM medium containing 30 ng/mL M-CSF for 3 days, and then cultured in α-MEM medium containing 30 ng/mL M-CSF and 50 ng/mL RANKL for 48 h to induce cells to differentiate into osteoclasts (OCs).

### Viability and TRAP activity assay and TRAP staining of OCs

OCs induced from BMM cells in 96-well plates were treated with various concentrations of JTG for 48 h. The viability of OCs was measured with a MTT kit, and the TRAP activity was determined according to our previous methods. The TRAP activity was represented as nanomoles p-nitrophenol per minute per 100 OCs. For TRAP positive multinucleated cell staining, BMMs-derived OCs were fixed with 4% paraformaldehyde and stained for TRAP activity using an Acid Phosphatase Kit (Sigma-Aldrich, St. Louis, MO, USA). The number and area of OCs containing 3 or more nuclei were counted and photographed using an inverted microscope (Nikon Corporation, Tokyo, Japan).

### The staining of F-actin ring of osteoclasts

OCs induced from BMM cells were seeded in confocal culture dishes (diameter, 35 mm), and treated with various concentrations of JTG for 48 h. BMMs-derived OCs were fixed with 4% paraformaldehyde for 20 min and then permeabilized with 0.1% Triton X-100 for 5 min. Then cells were washed and incubated with fluorescein isothiocyanate (FITC)-phalloidin for 45 min followed by 4′,6-diamidino-2-phenylindole (DAPI, Sigma Aldrich) staining for 10 min. Then, cells were washed and photo-graphed using a laser scanning confocal microscope (Meridian Co., USA).

### The analysis of expression of protein associated with osteoclast differentiation and activities

BMMs were plated in 6-well plates at a density of 2 × 10^6^ cells per well and induced by concentrations of 30 ng/mL M-CSF and 50 ng/mL sRANKL for 48 h. The BMMs-derived OCs were treated with various concentrations of JTG for 4 h, and then, were stimulated with 200 ng/mL LPS for 5, 15, 30, 60 min. Finally, the cells were washed with PBS and lysed in RAPI assay buffer for 30 min at 4 °C. The cell protein was collected and their contents were detected by BSA kits. The western blot analysis was used to determine the expression of proteins.

### Western-blot analysis

The cell lysates were incubated with a sample buffer of 5 × Laemmli at 100 °C for 5 min. An equal amount of protein was separated by 10% SDS-PAGE and transferred to the PVDF membrane. The membrane was then blocked with 5% BSA for 2 h and incubated with corresponding primary antibodies overnight at 4 °C, and then washed three times with TBST, incubated with the HRP-conjugated secondary antibody for 1 h at room temperature. The protein bands were detected with electro-chemiluminescence (ECL) reagent, and scanned using a chemiluminescence digital imaging system.

### Statistical analysis

All data analyses were completed using Graphpad Prism version 5.0 software and IBM SPSS statistic 22.0 software. Data are expressed as the mean ± SD. Data comparison was shown by using one-way analysis of variance (ANOVA). *P* values lower than 0.05 were considered statistically significant.

## Results

### The amino acid composition of peptides and proteins in JTG

**Fig. 1:**
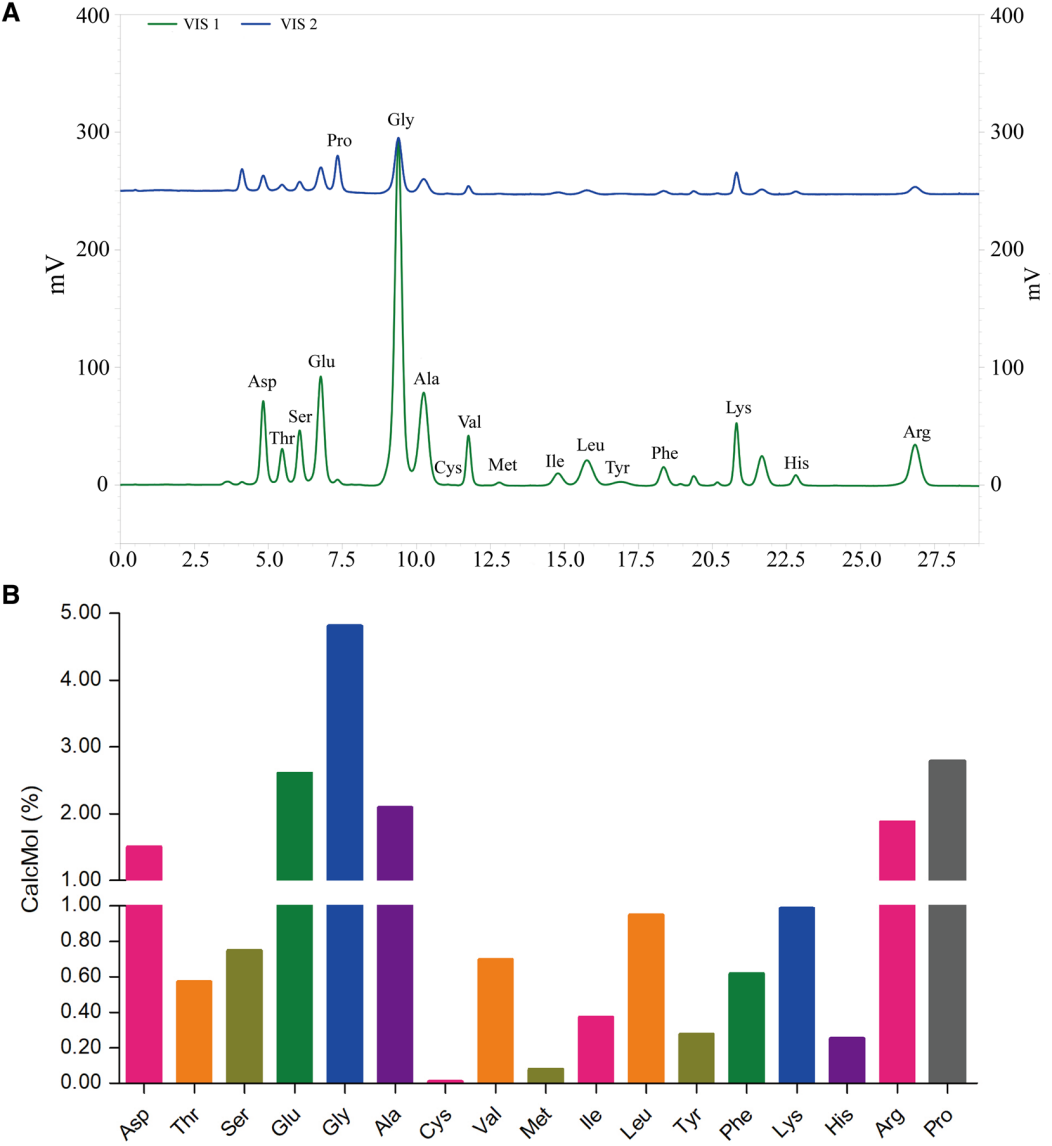
Determination of amino acid contents in JTG. **A** Amino acid analysis chromatogram in JTG degradation products; **B** Percentage of amino acids in JTG degradation products

The samples of JTG were treated to hydrolyze the peptides and proteins into amino acid, and the content of amino acid in JTG were determined by using Hitachi L-8900 Amino Acid Analyzer. As shown in Fig. 1A and B, the results showed that the degradation products of JTG contains 17 amino acid, and their composition as follows: 1.50% Aspartate (Asp), 0.57% Threonine (Thr), 0.75% Serine (Ser), 2.61% Glutamic acid (Glu), 4.81% Glycine (Gly), 2.09% Alanine (Ala), 0.01% Cysteine (Cys), 0.70% Valine (Val), 0.08% Methionine (Met), 0.37% Isoleucine (Ile), 0.95% Leucine (Leu), 0.28% Tyrosine (Tyr), 0.62% Phenylalanine (Phe), 0.99% Lysine (Lys), 0.25% Histidine (His), 1.88% Arginine (Arg) and 2.78% Proline (Pro).

### JTG does not affect body and uteri weight in OVX rats

**Fig. 2:**
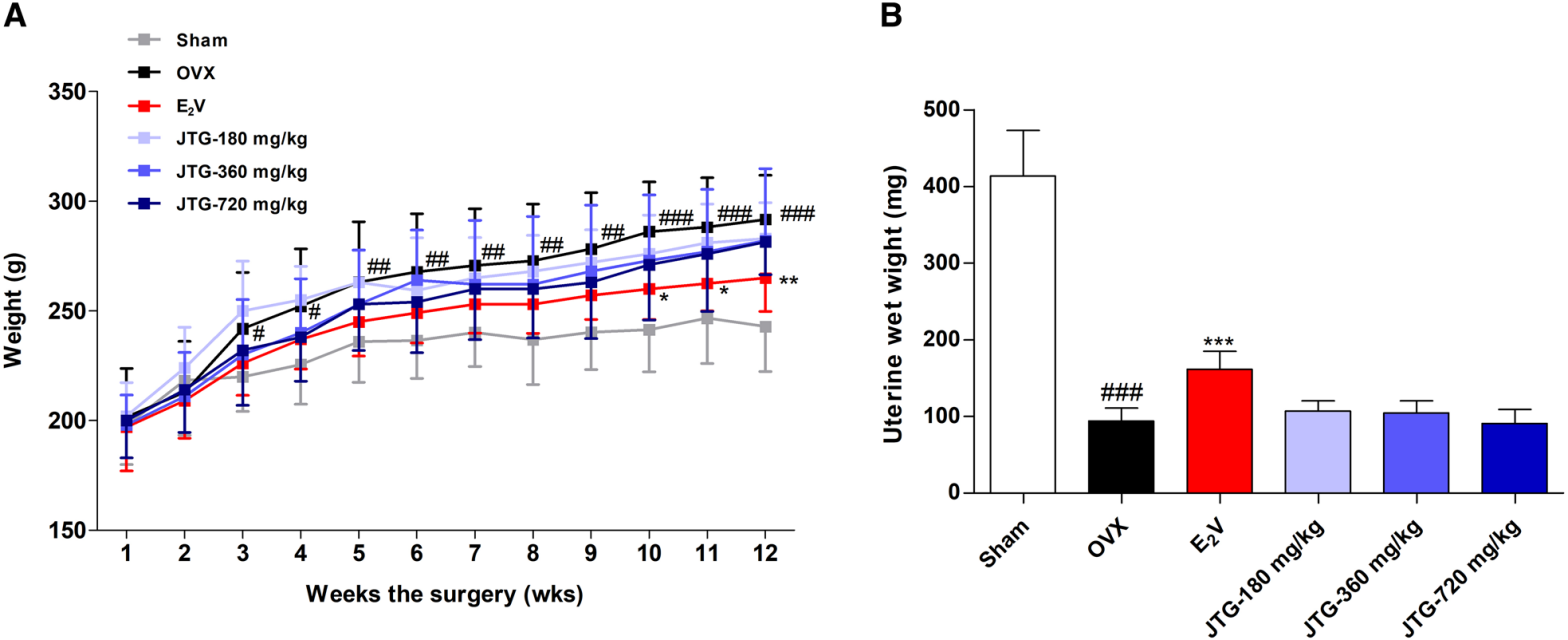
The effects of JTG on body weight (**A**) and uterine wet weight (**B**) of OVX rats. The data were expressed as mean ± SD (n = 10), ^#^*P* < 0.05, ^##^*P* < 0.01, ^###^*P* < 0.001, compared with Sham group; *P < 0.05, **P < 0.01, ***P < 0.001, compared with OVX group

As shown in Fig. 2A, the body weight of OVX rats were significantly increased compared to those of sham rats (*P* < 0.05, *P* < 0.01, *P* < 0.001), and administration with E_2_V significantly decreased the body weight of OVX rats (*P* < 0.05, *P* < 0.01), but administration of JTG had no significant effects on the body weight of OVX rats. Ovariectomy induces estrogen deficiency, leading to the uterine atrophy and decreased the uteri weight. As shown in Fig. 2B, the uterine wet weight of OVX rats was significantly decreased compared to those of sham rats (*P* < 0.001), and administration of E_2_V significantly increased the uterine wet weight (*P* < 0.001), but administration of JTG had no significant effects on the uterine wet weight of OVX rats.

### JTG modulates bone metabolism as evidenced by urine and serum biochemical parameters in OVX rats

**Fig. 3:**
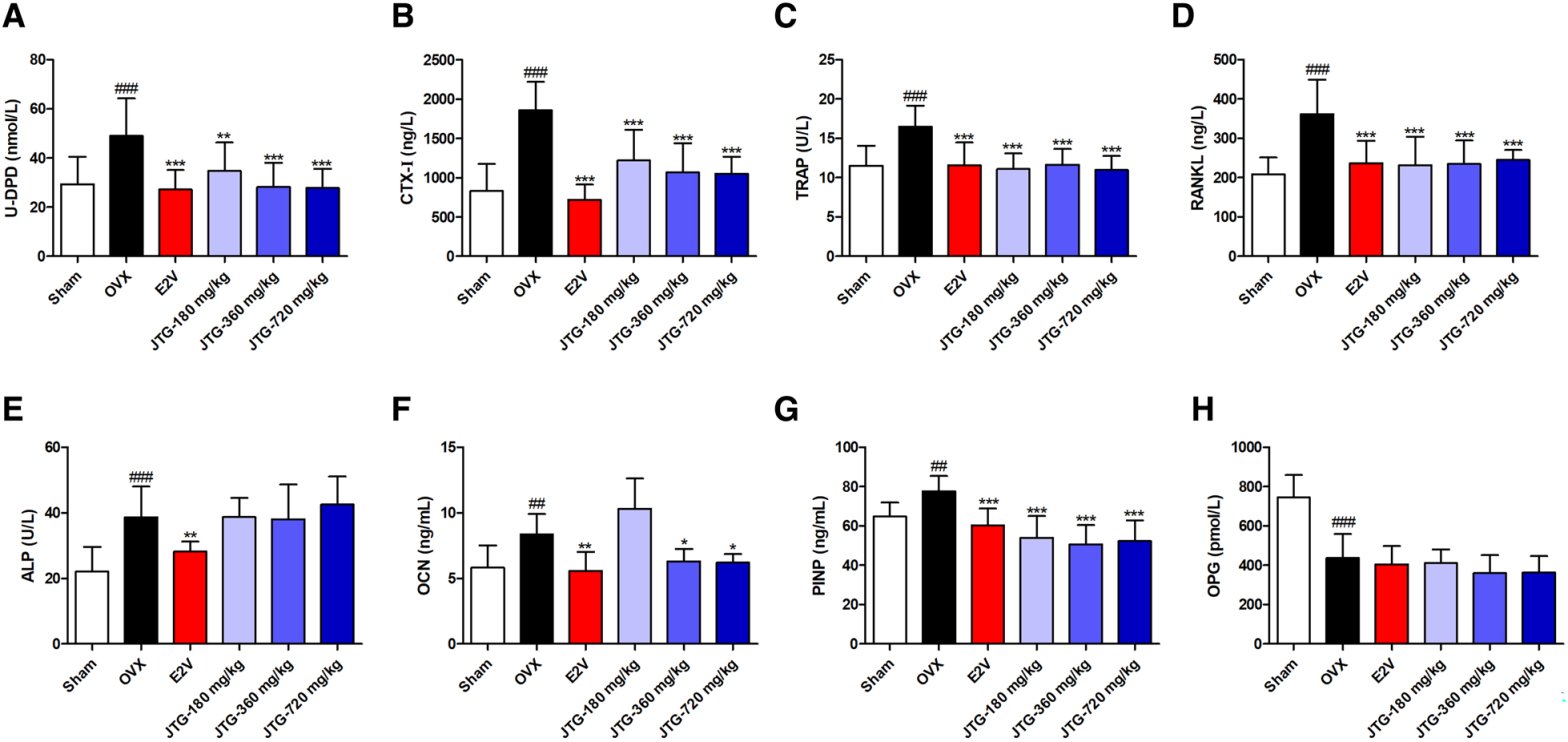
Effects of JTG on biochemical indicators in urine or serum of OVX rats. **A** Urine DPD; **B** serum CTX-I; **C** serum TRAP activity; **D** serum levels of RANKL; **E** serum ALP activities; **F** serum levels of OCN; **G** serum levels of PINP; **H** serum levels of OPG. The data were expressed as mean ± SD (n = 10), ^#^*P* < 0.05, ^##^*P* < 0.01, ^###^*P* < 0.001, compared with Sham group; *P < 0.05, **P < 0.01, ***P < 0.001, compared with OVX group

Ovariectomy cause bone loss, leading to increased levels of DPD, CTX-I and other degradation products of bone matrix in urine and serum of rats. As shown in Fig. 3A and B, the urine levels of DPD and serum levels of CTX-I in OVX rats were significantly elevated compared to those of sham rats (*P* < 0.001), and treatment with E_2_V and JTG significantly decreased the urine levels of DPD and serum levels of CTX-I in OVX rats (*P* < 0.01, *P* < 0.001).

Ovariectomy results in the high turnover of bone matabolism with characteristic of increase in both bone formation and bone resorption. Hence, the serum levels of ALP, OCN and PINP exhibiting activities of bone formation and serum levels of TRAP activities indicating activities of bone resorption were evaluated in OVX rats. As shown in Fig. 3C–G, the serum levels of ALP, OCN, PINP and TRAP were increased in OVX rats compared with those in sham rats (*P* < 0.01, *P* < 0.001), and treatment with E_2_V significantly decreased the levels of the above parameters in serum of OVX rats (*P* < 0.01, *P* < 0.001), and treatment with JTG significantly reduced the levels of OCN, PINP and TRAP in serum of OVX rats (*P* < 0.05, *P* < 0.01), but had no effects on ALP activities in serum of OVX rats.

RANKL and OPG exert an opposite effect on the differentiation of osteoclast. RANKL stimulates the differentiation of osteoclast, while OPG suppresses the differentiation of osteoclast. As shown in Fig. 3D and H, the serum levels of RANKL were increased (*P* < 0.001), and the levels of OPG were reduced in OVX rats (*P* < 0.001), treatment with E_2_V and JTG significantly decreased the serum levels of RANKL in OVX rats (*P* < 0.001), but had no effects on serum levels of OPG in OVX rats.

### JTG increases BMD and improves the micro-architecture of femurs in OVX rats

**Fig. 4:**
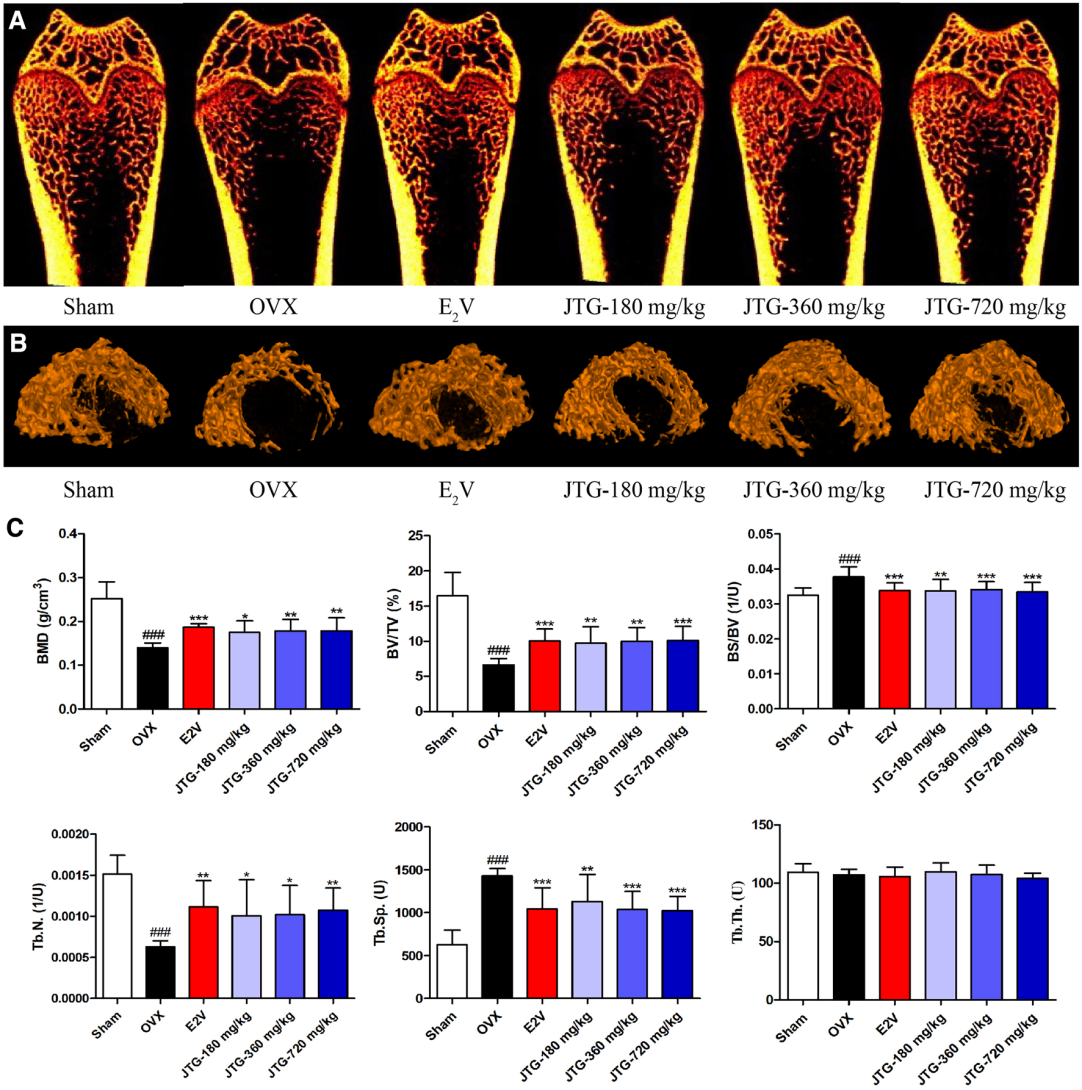
Effects of JTG on micro-architecture and histomorphometric parameters of bone tissue in the distal femur of OVX rats. **A** Represent 2D images of bone tissue scanned with Micro-CT; **B** Represent 3D scanning images of bone tissue scanned with Micro-CT; **C** The histomorphometric parameters of bone tissue analyzed by Micro-CT, including BMD, BV/TV, BS/TV, Tb.N, Tb.Sp and Tb.Th. The data were expressed as mean ± SD (n = 10). ^###^*P* < 0.001 compared with Sham group; *P < 0.05, **P < 0.01, ***P < 0.001 compared with OVX group

The osteoporosis of rats induced by ovariectomy is characterized by decreased BMD and deteriorated microarchitecture. The micro-CT analysis showed that trabecular bone became sparse, and had a bigger space in distal femur of OVX rats compared with those of sham rats, and treatment with E_2_V and JTG resulted in a more compacted trabecular bone in distal femur of OVX rats (Fig. 4A and B). The bone histomorphometric analysis indicated that the BMD, Tb.N and BV/TV were significantly decreased (*P* < 0.001), and BS/BV and Tb.Sp were significantly increased (*P* < 0.001), Tb.Th had no significant alteration in distal femur of OVX rats compared to those of sham rats, treatment with E_2_V and JTG caused an increase in BMD, Tb.N and BV/TV (*P* < 0.05, *P* < 0.01, *P* < 0.001), and a decrease in BS/BV and Tb.Sp (*P* < 0.01, *P* < 0.001), and had no effects on Tb.Th in distal femur of OVX rats (Fig. 4C). These results demonstrated that JTG could prevent OVX-induced bone loss and improve the bone microarchitexture.

### JTG improves the properties of bone biomechanics in the OVX rats

**Fig. 5:**
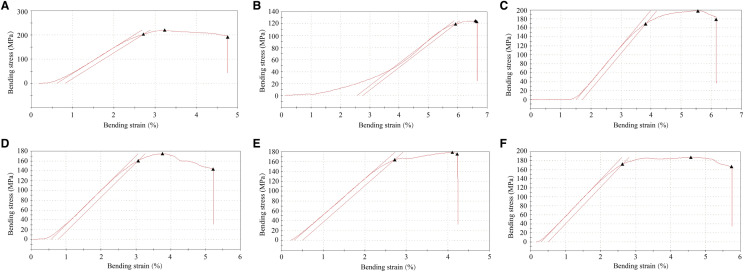
The stress–strain curves in three point bending test. **A** Sham group; **B** OVX group; **C** E_2_V group; **D** JTG-180 mg/kg group; **E** JTG-360 mg/kg group; **F** JTG-720 mg/kg group;

To estimate whether administration of JTG can improve bone strength in OVX rats, bone biomechanical properties were determined with three point bending test. As shown in Fig. 5 and Table 1, there were a significant decrease in elastic modulus, bending stiffness, maximum load, maximum stress and bone material toughness (*P* < 0.05, *P* < 0.01, *P* < 0.001), and no change of maximum strain of femur in OVX rats as compared to those of sham rats, and administration of JTG and E_2_V normalized these parameters, exhibiting that JTG could improve the bio-mechanical properties of bone tissue, and decrease the risk of bone fracture (*P* < 0.05, *P* < 0.01, *P* < 0.001).

**Table 1:** The effect of JTG on bone biomechanical parameters in OVX rat (n = 10, means ± SD)

Group	Simple size (mm)	Elastic modulus (MPa)	Maximum load (N)	Maximum stress (MPa)	Bending stiffness (N/mm^2^)	Maximum strain (mm/mm)	Material toughness (KJ)
Sham	3.15 ± 0.16	7723.82 ± 1218.10	143.49 ± 7.88	184.68 ± 23.71	35,207.68 ± 5452.17	0.058 ± 0.009	60.28 ± 6.24
OVX	3.08 ± 0.14	5419.00 ± 776.01^###^	126.88 ± 9.93^##^	153.59 ± 15.30^###^	27,506.02 ± 5241.84^###^	0.057 ± 0.013	48.63 ± 7.61^#^
E_2_V	3.14 ± 0.08	7944.48 ± 714.80***	146.10 ± 16.08***	189.70 ± 10.85***	35,612.25 ± 3178.47***	0.057 ± 0.006	56.76 ± 7.28
JTG-180 mg/kg	3.09 ± 0.14	6924.44 ± 764.04**	145.56 ± 9.67***	175.31 ± 12.85**	33,837.09 ± 3103.59**	0.059 ± 0.008	59.84 ± 6.65*
JTG-360 mg/kg	3.12 ± 0.13	7260.66 ± 765.78***	148.15 ± 13.46***	178.96 ± 13.71**	34,854.07 ± 3922.87**	0.057 ± 0.011	60.98 ± 9.67**
JTG-720 mg/kg	3.15 ± 0.18	7409.68 ± 1442.85***	148.30 ± 12.98***	180.75 ± 22.37***	35,384.66 ± 7091.08***	0.059 ± 0.006	60.63 ± 12.49*

^#^*P* < 0.05, ^##^*P* < 0.01, ^###^*P* < 0.001, compared with the Sham group

*P < 0.05, **P < 0.01, ***P < 0.001, compared with the OVX group

### JTG is involved into the regulation of BMP/Smad and Wnt/β-catenin signaling pathway in bone of OVX rats

**Fig. 6:**
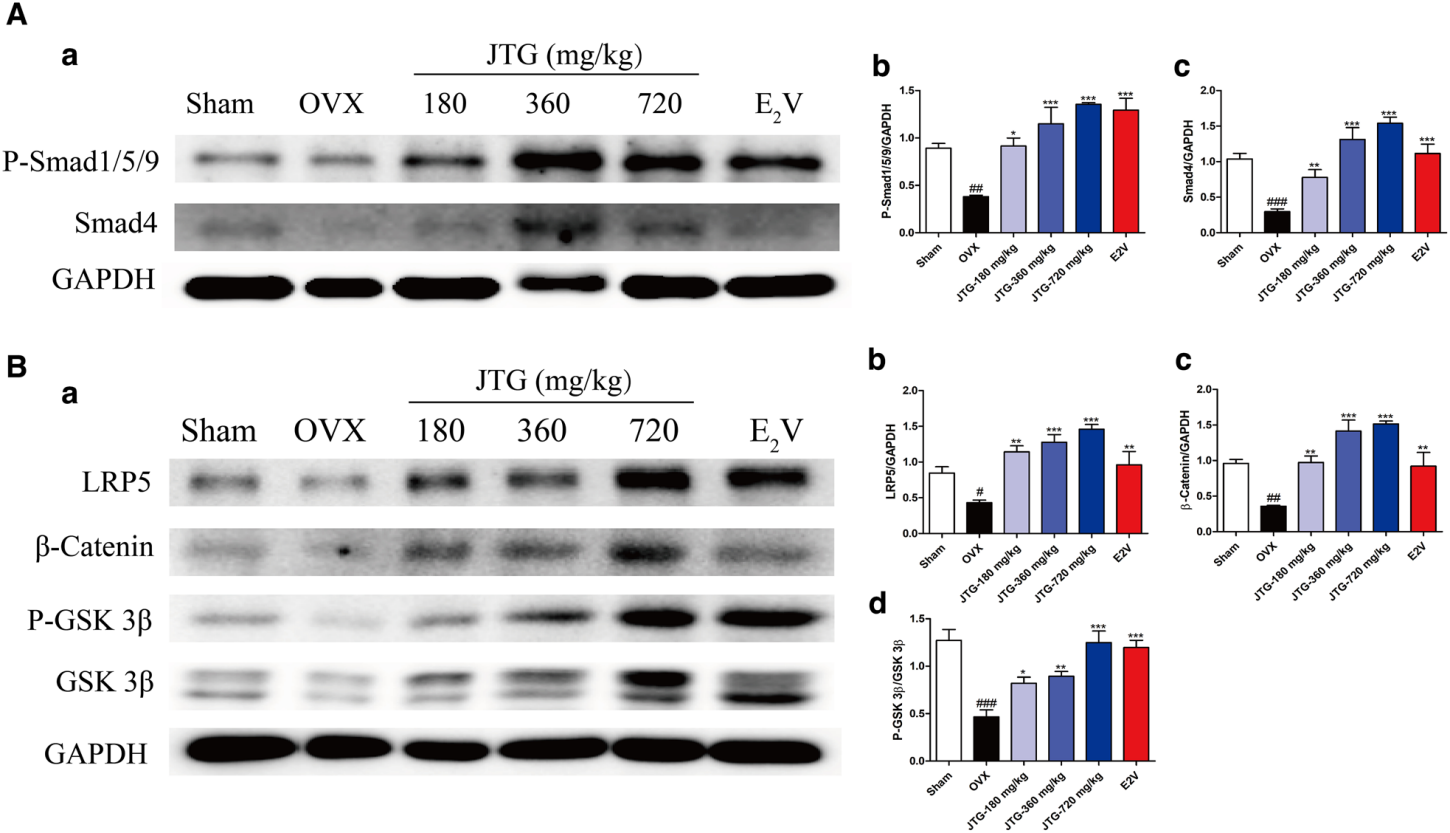
Effects of JTG on the expression of key proteins in BMP and Wnt/β-catenin pathway in bone tissue of OVX rats. The expression of protein in **A**: **a–c** BMP pathway and **B**: **a**–**d** Wnt/β-catenin pathway was analyzed by Western-blot. The data were expressed as mean ± SD (n = 4). ^#^*P* < 0.05, ^##^*P* < 0.01, ^###^*P* < 0.001 compared with Sham group; *P < 0.05, **P < 0.01, ***P < 0.001 compared with OVX group

BMP/Smad and Wnt/β-catenin signaling pathway are involved into the regulation of osteoblast differentiation. To explore the possible mechanism of JTG on osteogenesis, the expression of key proteins in BMP/Smad and Wnt/β-catenin signaling pathway were analyzed by using western blot method. As shown in Fig. 6A, Smad 4 and the phosphorylation of Smad1/5/9 in BMP pathway was significantly reduced in femurs of OVX rats compared with those of Sham rats (*P* < 0.01, *P* < 0.001), and administration of JTG and E_2_V significantly increased the expression of Smad 4 and the phosphorylation of Smad1/5/9 in femurs of OVX rats (*P* < 0.05, *P* < 0.01, *P* < 0.001). As shown in Fig. 6B, the expression of LRP5 and β-catenin and phosphorylation of GSK-3β in Wnt/β-catenin were significantly decreased in the femurs of OVX rats compared with those of Sham rats (*P* < 0.05, *P* < 0.01, *P* < 0.001), and administration of JTG and E_2_V significantly elevated expression of LRP5 and β-catenin (*P* < 0.01, *P* < 0.001), and promoted the phosphorylation of GSK-3β in the femurs of OVX rats (*P* < 0.05, *P* < 0.01, *P* < 0.001). These results implied that JTG maybe increased the bone formation by regulating BMP and Wnt/β-catenin pathway.

### JTG enhances osteoblast differentiation of BMSCs

**Fig. 7:**
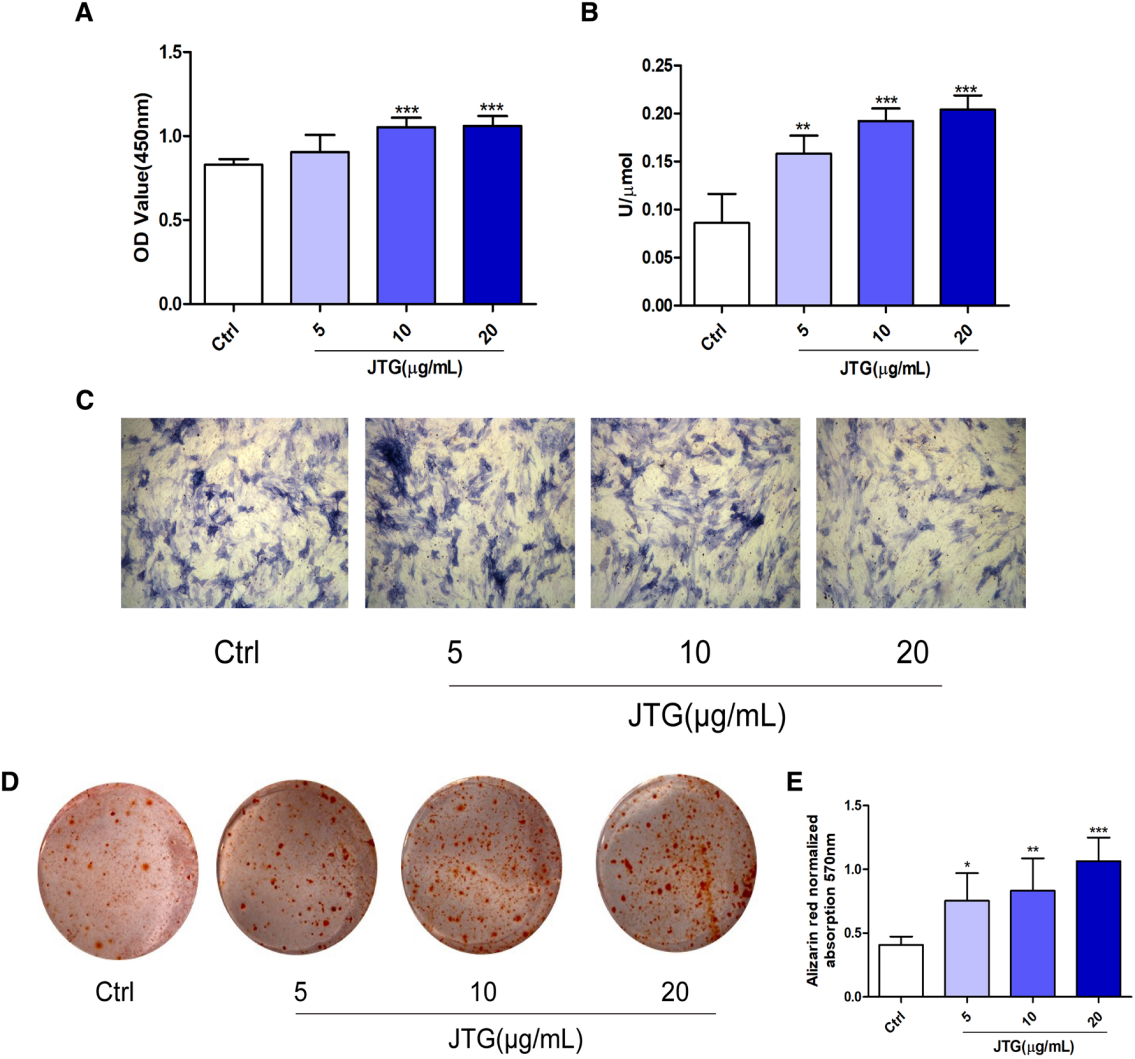
Effects of JTG on osteoblast differentiation of BMSCs. **A** The BMSCs were treated with JTG for 48 h, the cell proliferation was assayed by CCK-8 method. **B** and **C** The BMSCs were treated with JTG for 7 d, the ALP activities and staining were performed by using assay kits. **D** The BMSCs were treated with JTG for 21 d, and stained with alizarin red to observe the bone nodules under microscope (×10), and **E** the bone nodules were dissolved with 10% (V/W) cetylpyridinium phosphate chloride, and the absorbance was measured at 570 nm with a microplate reader to quantify cell mineralization. The data were expressed as mean ± SD (n = 4). *P < 0.05, **P < 0.01, ***P < 0.001 compared with control group

BMSCs have the potentials to differentiate into osteoblast, and then produce new bone matrix. As shown in Fig. 7, treatment BMSCs with JTG at dose of 5, 10 and 20 μg/mL significantly increased the proliferation and the activity of ALP, and promoted the formation of bone mineral nodules (*P* < 0.05, *P* < 0.01, *P* < 0.001), exhibiting that JTG significantly enhanced BMSCs to differentiate into osteoblast, and then increased the bone formation.

### JTG regulates BMP and Wnt/β-catenin signaling pathway in BMSCs

**Fig. 8:**
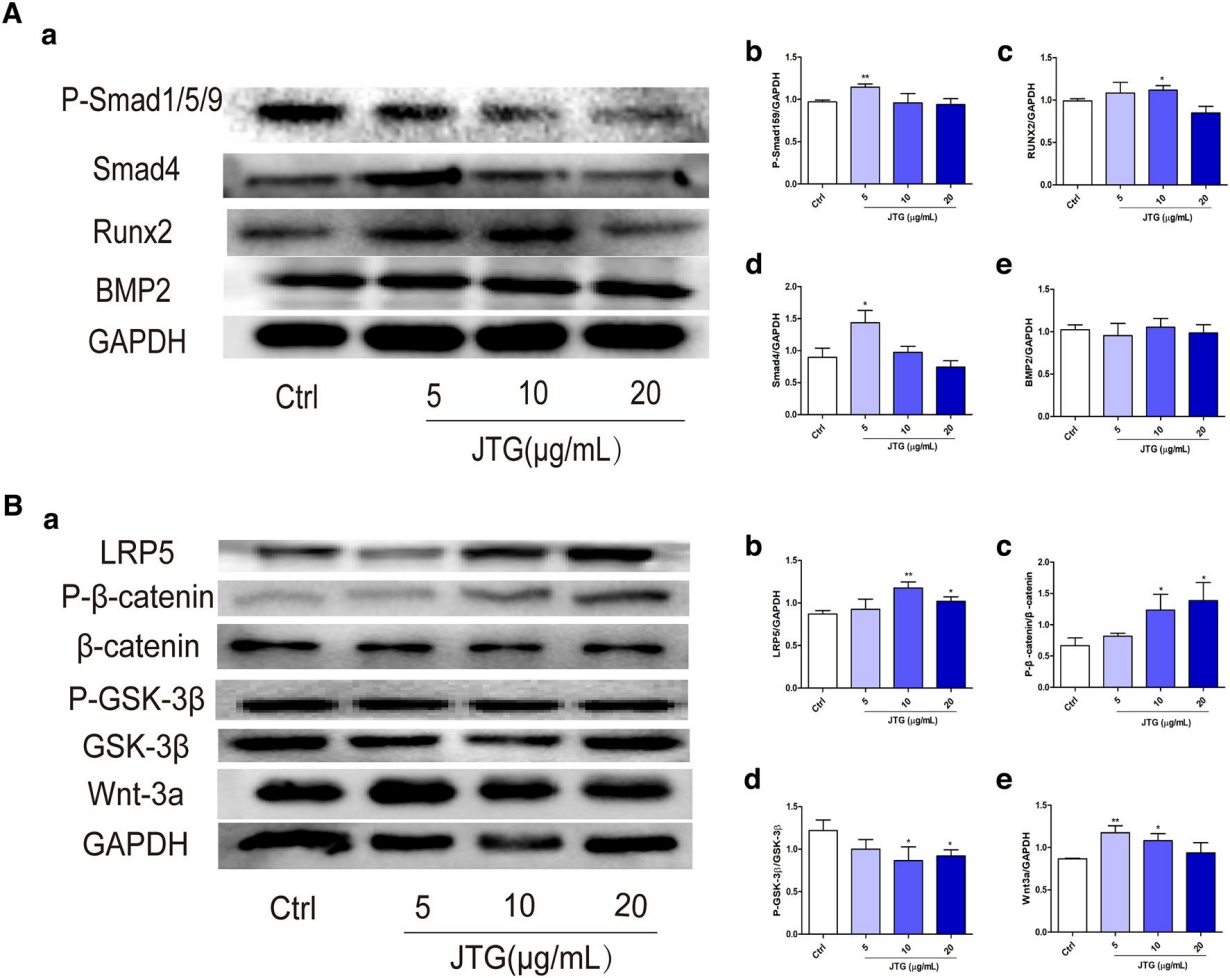
The regulatory effects of JTG on BMP and Wnt/β-catenin pathway osteoblast differentiation of BMSCs. The BMSCs were treated with JTG for 21d, the expression of proteins in (**A**: **a**–**e**) BMP and (**B**: **a**–**e**) Wnt/β-catenin pathway was analyzed with Western-blot. The data were expressed as mean ± SD (n = 3). *P < 0.05, **P < 0.01 compared with control group

JTG has been shown to modulate BMP and Wnt/β-catenin signaling pathway in the bone tissue of OVX rats. Further investigation was conducted to observe the regulatory effects of JTG on the above pathways in BMSCs. As shown in Fig. 8A, treatment with JTG at dose of 5, 10 and 20 µg/mL significantly increased the expression of P-Smad1/5/9, Smad4 and Runx2 in BMSCs (*P* < 0.05, *P* < 0.01), but did not exert significant effects on the expression of BMP2. As shown in Fig. 8B, treatment with JTG at dose of 5, 10 and 20 µg/mL significantly enhanced the expression of wnt-3a and LRP5 (*P* < 0.05, *P* < 0.01), increased the phosphorylation of β-catenin (*P* < 0.05), and inhibited the phosphorylation of GSK-3β in BMSCs (*P* < 0.05). These results demonstrated that JTG promoted the osteoblast differentiation of BMSCs by modulating BMP and Wnt/β-catenin pathways.

### JTG inhibits the formation and differentiation of osteoclast derived from BMM

**Fig. 9:**
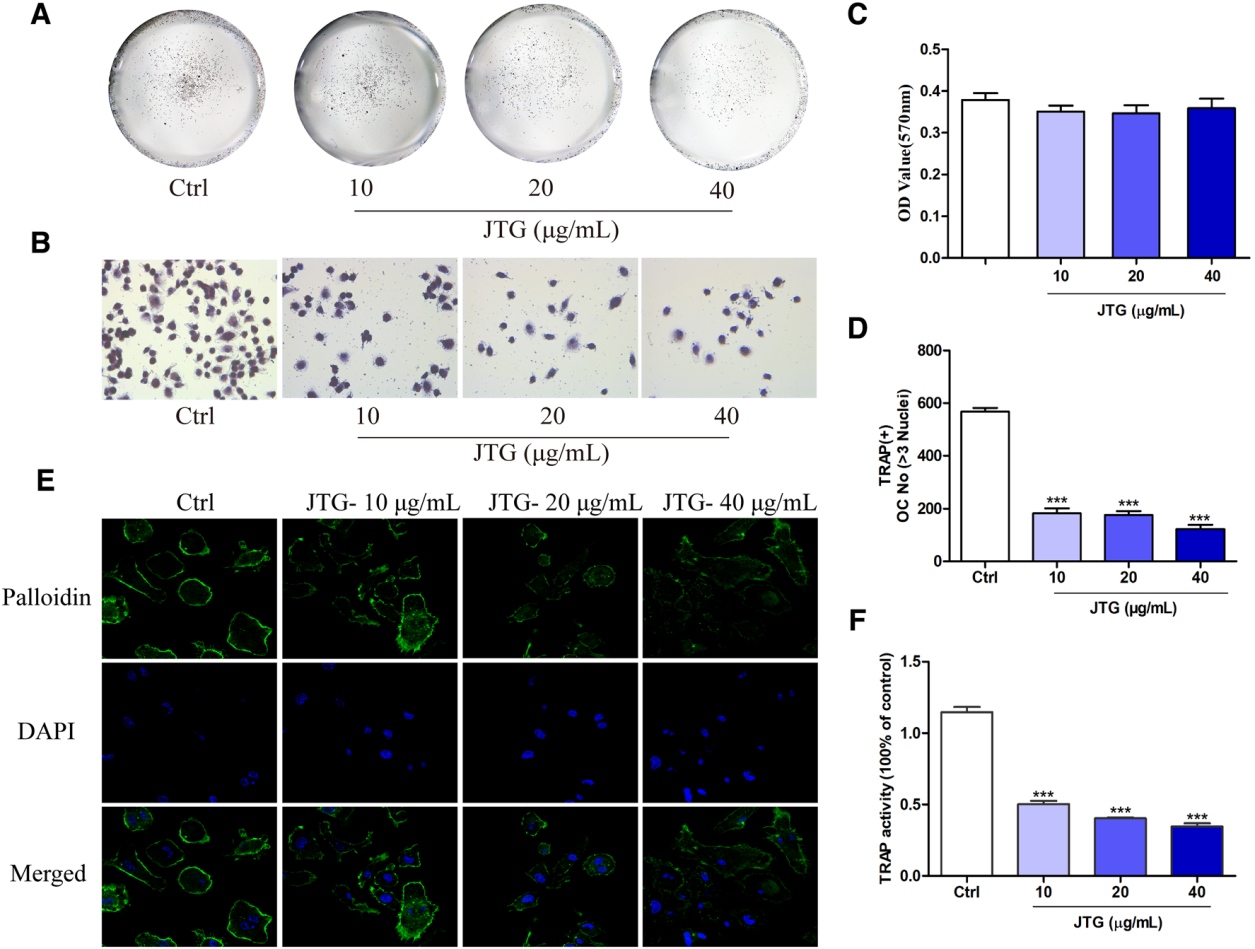
Effects of JTG on formation and differentiation into osteoclasts of BMMs. The osteoclasts were induced from bone marrow macrophages (BMMs) of the femur in C57BL/6 mice with RANKL and M-CSF. **A** and **B** TRAP staining for osteoblast treated with JTG at concentration of 10, 20 and 40 μg/mL for 3 days. **C** Viability of osteoclasts treated with 10, 20 and 40 μg/mL JTG for 48 h was assessed by the MTT kits. **D** The number of TRAP-positive multinucleated osteoclast treated with JTG for 3 days. **E** F-actin rings of osteoclast treated with JTG for 48 h were stained with phalloidin and DAPI respectively, and then imaged with a fluorescence microscope (×630). **F** TRAP activities of osteoclast treated with JTG measured by *p*-nitrophenyl sodium phosphate method. The results are expressed as means ± SD (n = 6). The experiments were repeated for three times. *P < 0.05, **P < 0.01 compared with control group

Osteoclasts are the unique cells that perform bone resorption. We investigated the effects of JTG on formation and differentiation of osteoclast derived from BMMs. As shown in Fig. 9A–D and F, JTG at concentration of 10, 20, and 40 µg/mL did not affect the vitality of BMMs, and decreased the numbers of TRAP positive osteoclast and the TRAP activities of osteoclast derived from BMMs with RANKL (*P* < 0.001). The F-actin ring, as an essential bone resorption structure in OCs, were visualized by the phalloidin fluorescent staining. As shown in Fig. 9E, treatment with JTG at the concentrations of 10, 20 and 40 µg/mL suppressed the construction of the F-actin ring in OCs, as evidenced by their thickness and completeness.

### JTG suppresses the activation of NF-κB pathway in osteoclast derived from BMM

**Fig. 10:**
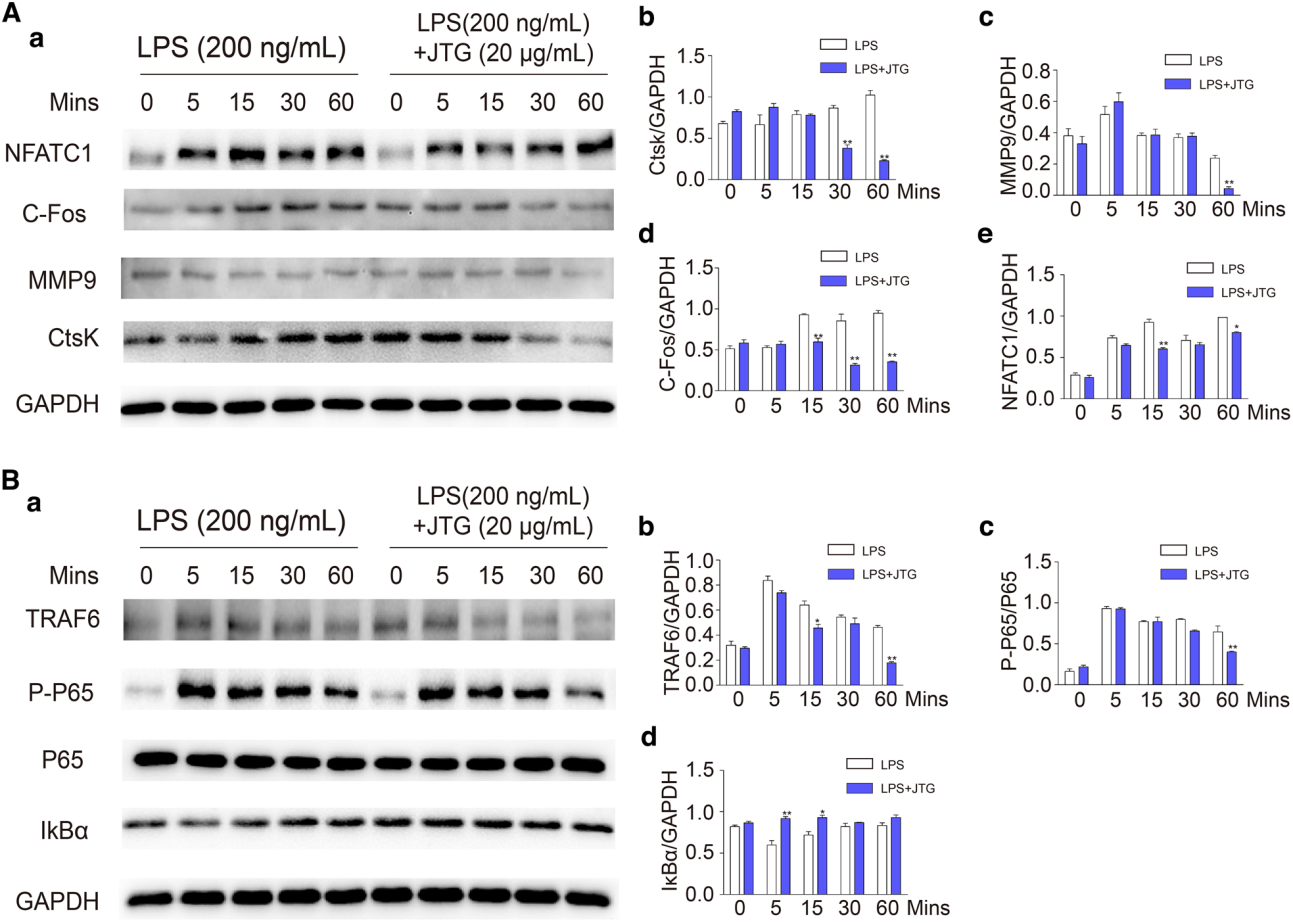
Effects of JTG on expression of associated proteins and NF-κB pathway of osteoclast induced from BMMs with RANKL and LPS. BMMs were incubated with RANKL and JTG for 48 h, the proteins were extracted to analyze associated proteins of osteoclast by Western blot. **A**: **a** Western blot imagines for expression of NFATc1, c-Fos, Cathepsin K and MMP9. **A**: **b**–**e** The quantification analysis of NFATc1, c-Fos, Cathepsin K and MMP9 based on the results of **A**: **a** by ECL detection system, respectively. **B**: **a** The images of Western blot for TRAF6, P-P65, P65 and IκBα. **B**: **b**–**d** The quantification analysis of TRAF6, P-P65/P65 and IκBα based on the results of **B**: **a** by using an ECL detection system, respectively. Each point represents the mean ± SD (n = 3). The experiments were repeated for three times. *P < 0.05, **P < 0.01 compared with control group

Firstly, the osteoclast differentiation is regulated by transcription factor NFATC1 and C-Fos, and MMP9 and CtsK participates in the degradation of bone minerals and collagen, respectively. The effects of JTG on these proteins in OCs were investigated using Western-blot. As shown in Fig. 10A, treatment OCs with JTG reduced the expression of NFATC1, C-Fos, MMP9, and Ctsk (*P* < 0.05, *P* < 0.01), further demonstrating that JTG inhibited the differentiation and function of OCs.

Moreover, NF-κB signaling pathway is involved into the regulation of mature and differentiation of OCs, so we investigated the regulatory effects of JTG on NF-κB signaling pathway of OCs. As shown in Fig. 10B, JTG inhibited the phosphorylation of P65, and the accumulation of TRAF6, and promoted the expression of IκBa in the NF-κB pathway of OCs (*P* < 0.05, *P* < 0.01), indicating that JTG inhibited the activation of NF-κB signaling pathway of OCs, and then decreased the osteoclastic bone resorption.

## Discussion

This study was designed to investigate the anti-osteoporotic potentials of JTG in ovariectomized rats, osteoblast derived from BMSCs of rats and osteoclast derived from BMM of mice. The evaluation of the treatments was done with histologic, biochemical, and analyses of regulatory protein expression of bone and bone mineral density, in addition to further verification in osteoblast and osteoclast. The findings demonstrated that JTG could exerts significantly bone protective effects through increasing bone formation by involving the regulation of BMP and Wnt/β-catenin pathways, and diminishing bone resorption via modulating NF-κB pathway.

Ovariectomy results in estrogen deprivation, and then leading to a significantly increased body weight and decreased uterine wet weight in comparison with the intact animals [19, 31, 32]. Administration of E_2_V caused a reduction of body weight and an increase of uteri weight in OVX rats, this implied a potential risk of incidence of endometrial cancer [33–35]. Treatment with JTG did not exert any effects on body and uterine weight of OVX rats, this lack of stimulating activity on uterine hyperplasia of JTG may be beneficial in reducing the risk of endometrial cancer and other disease associated with estrogen treatment.

The risk of bone fracture of osteoporotic patients is associated with a decrease in trabecular bone density and a deterioration of the bone micro-architecture, with a particular diminution in the total number of trabecular and an increase in the number of their perforations [36]. The analysis of Micro-CT found that bone loss in the OVX rats was increased as assessed by BMD and the number of trabecular [37]. The BMD and trabecular number were increased, trabecular thickness was not altered, and trabecular separation was decreased in the JTG treated OVX rats, suggesting that enhanced bone mineral density by JTG might be due to the increase in trabecular number, but not the trabecular thickness. Several clinical trials have demonstrated that JTG could improve the BMD in osteoporotic patients [38–40]. The effects of increasing BMD of JTG in OVX rats were collaborated with the inhibiting effects on the levels of urine DPD and serum CTX-I, and the results of clinical trials also further confirming the antiosteoporotic activities of JTG.

The bone biomechanical properties are often used to evaluate the bone quality and the risk of bone fracture. The three point bending test demonstrated that the ovariectomy decreased the biomechanical properties, and JTG treatment improved biomechanical properties of the femurs in OVX rats as evidenced by elastic modulus, maximum load, maximum stress, bending stiffness and bone material toughness. Furthermore, the correlation analysis of the BMD and bone biomechanical parameters showed that BMD of rats in each group was positively related to the bone bio-mechanical parameters of maximum stress, maximum load, elastic modulus and bending stiffness (Additional file 1: Table S1), indicating that the change of the BMD affected the bone biomechanical performance, and the improvement effects of JTG on the bio-mechanical properties of bone tissue also attributed to decreased risk of bone fracture.

Ovariectomy induces estrogen deficiency, and then bone loss and osteoporosis, which is similar to the pathological features of postmenopausal osteoporosis [41, 42]. It has been reported that long-term estrogen deficiency increased both bone resorption and bone formation in postmenopausal women, suggestive of enhanced bone turnover with increased net bone loss [43]. The biochemical markers associated with bone metabolism are widely used to assess bone-related disorders [44, 45]. The present study found that biomarkers for bone formation, such as serum levels of ALP, OCN and PINP, and the biomarkers for bone resorption, such as serum levels of RANKL and TRAP activities were elevated in OVX rats, and treatment with JTG reduced the serum levels of OCN, PINP and TRAP. The clinical trials also showed that JTG reduced the TRAP activities and levels of RANKL, and elevated the levels of OCN in serum of osteoporotic patients. Our findings, together with clinical trials revealed that that JTG reversed the bone high turnover in OVX rats and decreased bone loss by modulating osteoblastic bone formation and osteoclastic bone resorption.

Osteoblasts, which produce bone matrix proteins and differentiate into osteocytes, are derived from undifferentiated mesenchymal cells [46]. The differentiation from progenitor cells to osteoblast is controlled by BMP and Wnt/β-catenin pathways. BMPs signaling is involved in the induction of osteogenic differentiation and regulation of bone formation [47]. Generally, BMP binds with its receptor to activate protein of Smad1/5/8, and then works in conjunction with Osterix via both Runx2 dependent and independent pathways to induce osteogenesis [48]. BMP-2, the most commonly studied BMP ligand, induces MSC osteogenesis both in vitro and in vivo [49]. Wnt signaling pathway is activated by the binding of the ligands such as Wnt1 and Wnt3a to single-pass transmembrane co-receptor LRP5/6, and also mediated by β-catenin [50]. In the absence of Wnt stimulation, cytoplasmic β-catenin is phosphorylated by GSK-3β, and phosphorylated β-catenin is further ubiquitinated and rapidly degraded by the proteasomal system to prevent cytoplasmic accumulation [51]. On the other hand, Wnt stimulation suppresses GSK-3β activity and induces the cytoplasmic accumulation of β-catenin. The accumulated β-catenin translocates to the nucleus where it induces the expression of target genes to induce osteogenesis of BMSCs [52, 53]. The Western-blot analysis of bone tissue and osteoblast derived from BMSCs revealed that treatment with JTG enhanced the expression of protein in the pathway of BMP and Wnt/β-catenin pathway, indicating that JTG improved bone formation maybe through involving into the regulation of BMP and Wnt/β-catenin pathway. The effects of JTG on differentiation of BMSCs into osteoblast verified our findings as assessed by proliferation, ALP activity and mineralization of bone matrix.

The osteoclast, the functional cells of resorbing bone matrix, are derived from BMMs, and the osteoclastgenesis of BMMs are stimulated by macrophage-colony stimulating factor (M-CSF) and receptor activator of nuclear factor-κB ligand (RANKL) and modulated by NF-κB pathway [54]. The modulation of RANKL on osteoclast differentiation is mediated by tumor necrosis factor receptor associated factor 6 (TRAF6), and then leading to the activation of MAPKs and NF-κB. Activated NF-κB induces the expression of NFATc1 and c-Fos. Finally, NFATc1 translocates into the nucleus, and induces the expression of osteoclast-specific target genes, such as CK and MMP-9 [54, 55]. JTG inhibited the expression of NFATc1, C-Fos, MMP9, and Ctsk, and also suppressed the phosphorylation of P65, and the accumulation of TRAF6, and enhanced the expression of IκBα in the NF-κB pathway, indicating that JTG decreased the osteoclastic bone resorption by inhibiting the activation of NF-κB signaling pathway of OCs.

JTG were prepared from several farmed animal skeleton, containing 18% calcium, 8% phosphorus, and abundant peptides and proteins, and the peptides and proteins are degraded into amino acid in vivo. Firstly, the deficiency of Ca is the main risk factor for osteoporosis, and conversely, increased dietary Ca intake improves bone mineral density [56]. Calcium is a fundamental bone mineral, and also influences many extracellular and intracellular processes, including development, growth, and maintenance of bone, and the stability of the cytoskeleton [57, 58]. Phosphorus is a macroelement involved in many biological processes, and also the bone building materials. High P levels inhibited osteoclast differentiation and activity [59]. Secondly, the proteins accounts for approximately 50% of bone volume and about a third of bone mass. Higher protein intakes are beneficial in attenuating age-related bone loss and reducing hip fracture risk in older subjects [60, 61]. Thirdly, a specific amino acid profile correlates with greater BMD and lower subsequent fracture risk. The higher serum valine, leucine, isoleucine and tryptophan concentrations were associated with less hip BMD decline [62]. Certain amino acid types, like members of the aromatic amino acid, can potently stimulate increases in intracellular calcium and extracellular regulated protein kinases (ERK) phosphorylation/activation [63]. Arginine enhances osteogenesis in human mesenchymal stem cells through upregulation of expression of osteogenic transcription factors [64]. Glutamic acid can significantly increase osteoblast differentiation and mineralization [65]. The degradation products of JTG had the high levels of Asp, Glu, Gly, Ala, Tyr, Phe, Arg and Pro, which may lead to an increased bone formation and a decreased bone resorption, and then reduced the bone loss in ovariectomized rats. Therefore, it is speculated that the calcium, phosphorus, peptides and proteins may be together attribute to anti-osteoporotic effects of JTG.

## Conclusion

The present study demonstrated that JTG treatment prevented bone loss and the deterioration of bone microarchitecture in ovariectomy induced rat model of osteoporosis by increasing bone formation and decreasing bone resorption. Furthermore, JTG also promoted the differentiation of BMSCs towards osteogenesis and inhibited differentiation of BMM towards osteoclastogenesis. Therefore, our investigation maybe provide further evidence for JTG being applied to prevent and treat osteoporosis in clinics.

## Supplementary Information

**Additional file 1: Table S1.** The correlation coefficient and coefficient T test between BMD and bone biomechanics.

## Data Availability

All datasets used and/or analyzed during the current study are available from the corresponding author on reasonable request.

## Abbreviations

α-MEM: α-Modified minimal essential medium
ACN: Acetonitrile
ALP: Alkaline phosphatase
BMD: Bone mineral density
BMMs: Bone marrow macrophages
BMPs: Bone morphogenetic proteins
BMSC: Bone marrow mesenchymal stem cell
BS/BV: Bone surface to bone volume
BVF: Bone volume fraction
Ca: Calcium
CtsK: Cathepsin K
CTX: C-Telopeptide of type I collagen
DPD: Deoxypyridinoline
ELISA: Enzyme linked immunosorbent assay
E_2_V: Estradiol valeratse
GSK-3β: Glycogen synthase kinase-3 β
JTG: Jin-Tian-Ge
LPS: Lipopolysaccharides
MMP9: Matrix metallopeptidase 9
NFATc1: Nuclear factor of activated T-cells cytoplasmic 1
OCs: Osteoclasts
OCN: Osteocalcin
OPG: Osteoprotegerin
OVX: Ovariectomy
PBS: Phosphate buffered saline
PINP: Procollagen I N-terminal propeptide
RANKL: Receptor activator of nuclear factor kappa B ligand
Tb.N: Trabecular number
Tb.Sp: Trabecular separation
Tb.Th: Trabecular thickness
TRAP: Tartrate resistant acid phosphatase
