# Supplementary material for: Jin-Tian-Ge ameliorates ovariectomy-induced bone loss in rats and modulates osteoblastogenesis and osteoclastogenesis in vitro
Source: Chin Med. 2022 Oct 5;17:78. doi: 10.1186/s13020-022-00627-2 (PMC9533506; doi:10.1186/s13020-022-00627-2)
Supplement: Supplementary file 1 — Additional file 1: Table S1. The correlation coefficient and coefficient T test between BMD and bone biomechanics. [file 13020_2022_627_MOESM1_ESM.docx]

Additional file 1: Table S1. The correlation coefficient and coefficient T test between BMD and bone biomechanics

| Group | Elastic modulus/BMD | | Maximum load/BMD | | Maximum stress/BMD | | Bending stiffness/BMD | |
| --- | --- | --- | --- | --- | --- | --- | --- | --- |
|  | r | *P-*value | r | *P-*value | r | *P-*value | r | *P-*value |
| Sham | 0.7710 | 0.0251 | 0.7835 | 0.0214 | 0.7695 | 0.0256 | 0.8918 | 0.0029 |
| OVX | 0.7789 | 0.0227 | 0.8558 | 0.0067 | 0.8191 | 0.0129 | 0.5035 | 0.2038 |
| E2V | 0.7899 | 0.0197 | 0.4457 | 0.2684 | 0.6654 | 0.0717 | 0.7639 | 0.0274 |
| JTG-180 mg/kg | 0.7996 | 0.0172 | 0.7321 | 0.0389 | 0.7443 | 0.0342 | 0.6102 | 0.1080 |
| JTG-360 mg/kg | 0.7356 | 0.0375 | 0.8874 | 0.0033 | 0.7448 | 0.0340 | 0.7705 | 0.0253 |
| JTG-720 mg/kg | 0.8507 | 0.0074 | 0.8327 | 0.0103 | 0.7466 | 0.0333 | 0.7579 | 0.0293 |
